# Individual and household level factors associated with presence of multiple non-communicable disease risk factors in Kenyan adults

**DOI:** 10.1186/s12889-018-6055-8

**Published:** 2018-11-07

**Authors:** Frederick M Wekesah, Loise Nyanjau, Joseph Kibachio, Martin K Mutua, Shukri F Mohamed, Diederick E Grobbee, Kerstin Klipstein-Grobusch, Christine Ngaruiya, Tilahun N Haregu, Gershim Asiki, Catherine K Kyobutungi

**Affiliations:** 1 African Population and Health Research Center, 2nd Floor, APHRC Campus, Manga Close, Off Kirawa Road, Kitisuru, P.O. Box 10787 00100, Nairobi, Kenya; 2 Julius Global Health, Julius Center for Health Sciences and Primary Care, University Medical Center, Utrecht University, Utrecht, Netherlands; 3 Division of Non Communicable Diseases, Ministry of Health, Nairobi, Kenya; 4 The Institute of Global Health, Faculty of Medicine, University of Geneva (UNIGE), Geneva, Switzerland; 5 Division of Health Sciences, Warwick Medical School, University of Warwick, Coventry, UK; 6 Division of Epidemiology and Biostatistics, School of Public Health, Faculty of Health Sciences, University of the Witwatersrand, Johannesburg, South Africa; 7 Yale School of Medicine, New Haven, CT USA

**Keywords:** Non-communicable disease, Multiple risk, STEPs, Kenya

## Abstract

**Background:**

Non-communicable diseases (NCDs), are increasing globally, causing about 60% of disability-adjusted life years and 39.8 million deaths in 2015. Risk factors often cluster and interact multiplicatively in an individual and this is strongly associated with the development and severity of NCDs. We assessed the sociodemographic factors associated with the presence of multiple NCD risk factors among individuals aged 18 years and older in the Kenyan population.

**Methods:**

We used national representative data from 4066 individuals out of 4500 who participated in the WHO STEPs study in 2015. NCD risk factor counts were derived by summing the risk factors present in an individual and categorising into 1–3, 4–6 and 7+ risk factors in any combination of the 12 assessed NCD risk factors (hypertension, diabetes mellitus, cholesterol, insufficient physical activity, excessive alcohol use, tobacco use and obesity, excess sugar intake, insufficient fruit and vegetables intake, high salt consumption, and use of unhealthy cooking fats and oils). Ordered logistic regression was used to investigate the sociodemographic factors associated with an individual possesing multiple NCD risk factors.

**Results:**

Majority (75.8%) of the individuals in the study possesed 4–6 and 10% had ≥7 NCDs risk factors. Nearly everyone (99.8%) had insufficient fruits and vegetable intakes, 89.5% consumed high salt in their diet and 80.3% did not engage in sufficient physical activity. Apart from NCD risk count which increased with age among both men and women, associations with other socio-demographic factors differed between men and women. A woman of Akamba ethinicity had lower odds (0.43) while Meru women had higher odds (3.58) of higher NCD risk factor count, compared to the Kalenjin women. Among men, being a Kisii or Luo was associated with lower odds (0.48 and 0.25 respectively) of higher NCD risk factor count. Women in a marital union had higher odds (1.58) of a higher NCD risk factor count.

**Conclusion:**

Majority of Kenyan adults possess more than four NCD risk factors; a clear indication of an emerging epidemic of NCDs in this population. Effective and multi-sectoral interventions targeting multiple risk factors in individuals are required to mitigate especially the behavioural and modifiable NCD risk factors in Kenya.

## Background

Non-communicable diseases (NCDs), comprising of cardiovascular diseases (CVDs), diabetes, cancers and chronic pulmonary diseases are currently a major contributor to the burden of disease and mortality worldwide, with predictions showing that by the year 2020, NCDs will account for seven out of every ten deaths in developing countries, surpassing communicable diseases as the leading cause of death [1–3].

Although there exist few reliable data on the prevalence and distribution of NCDs and their risk factors in sub-Saharan Africa SSA [4], NCDs are expected to overtake infectious diseases as major sources of morbidity and mortality by the year 2035 in the region [4]. It is estimated that deaths from NCDs will rise from one in four of all deaths in 2004 to about 46% of all deaths in 2030 [5–7]. Currently, SSA reports a million annual deaths due to CVDs [8]. Findings of a recent review show prevalence of hypertension in SSA ranging from 27 to 34% in 2013 [9]. In 2017, it was estimated that about 15.9 million people aged 18–99 in SSA had diabetes (3.1% prevalence) with projections indicating that the number would increase by 156% to 41.6 million by the year 2045 (3.7% prevalence) [10]. In Kenya, more than half of all recent hospital deaths and almost 50% of all hospital admissions are attributable to NCDs [11].

The rising epidemic of NCDs in SSA is attributable to growing urbanisation, changing lifestyles, population growth, ageing and epidemiologic changes in the disease, also known as epidemiological transition [3, 12–16]. The epidemic is propagated by other socio-cultural and environmental factors such as changes in air quality and early childhood exposures to NCD risk factors [17, 18]. The likelihood of occurence of NCDs in an individual has been closely linked to the presence of four major modifiable behavioural risk factors: unhealthy diet, tobacco use, physical inactivity, and harmful alcohol use. These risk factors, which are well established, are also known to operate in a similar manner all over the world [19].

The World Health Organisation (WHO) has since prioritised action against these four behavioural risk factors globally [7]. A few studies have however shown that in some populations in Kenya, most people lack information and are unaware of NCD risk and the risk factors [20–22]. Other studies on the prevalence and distribution of NCD risk factors in Kenya are either sub-national or were conducted among sub-populations, and only focussed on specific NCD risk factors, mostly on diabetes and hypertension [23–28]. This study which used data from the first nationally representative survey on NCD risk factors in Kenya using a standardised chronic disease risk factors tool investigated the patterns and distribution of multiple behavioural and physiological risk factors for NCDs, together with their sociodemographic determinants in adult individuals in Kenya [29]. The study supports the efforts by WHO towards the prevention and control NCDs and their risk factors by answering to the call to ‘build the case for sustained action by estimating the burden of NCDs and their main risk factors’ by developing a national risk factor profile for NCDs [30]. Information on the national NCD risk factor profile could help predict and track the evolution of the NCD epidemic in Kenya, and can inform the design of targeted and effective multisectoral interventions at the policy, environmental and health system levels, to forestall the impending NCD epidemic in the country.

## Methods

### Data source, study participants and sampling

The WHO STEPs survey, carried out in Kenya between April and June 2015, employed a cross-sectional household study design that targeted adult individuals aged 18 years and older. A three-stage cluster sampling was used to, in the first stage, select 100 each of urban and rural clusters from the the fifth national sample surveys and evaluation programme (NASSEP V) sampling frame by the Kenya National Bureau of Statistics. In the second stage, a sample of 30 households in each cluster were identified, while in the third stage, one adult (aged between 18 and 69 years) from each household was randomly selected to participate in the study. Further details regarding the design of the study have been published previously [29].

### Data collection, measurements and definitions

Data was collected using personal digital assistants (PDA) loaded with the eSTEPS tool provided by the World Health Organisation [31]. Data was collected on the four main behavioural risk factors for NCDs (tobacco use, harmful use of alcohol, unhealthy diet and insufficient physical activity), as well as on the key physiological risk factors for NCD: overweight and obesity, raised blood pressure/hypertension, raised blood lipids and raised blood glucose/diabetes mellitus. Anthropometric measures for height and weight were also collected.

Blood pressure (BP) was diagnosed using a validated oscillometric automated digital BP device (OMRON™ digital automatic BP monitor). Using appropriate cuff sizes, three readings were taken on the left arm from a respondent in a seated position, at one minute intervals. The mean of the second and the third measurement was recorded. Raised blood pressure/hypertension was based on blood pressure readings cut-off for systolic > = 140 mmHg and/or diastolic > = 90 mmHg or if they reported to have been previously diagnosed and informed to have hypertension by a health care worker and/or were taking medication for raised blood pressure.

For a small number of respondents (84/4066), random blood sugar was taken because they did not fast as instructed. For all other respondents, fasting blood glucose measurements were taken a day after the first contact with the respondent, after they confirmed to have had an overnight fast. A drop of blood from a finger prick was used to test for glucose levels using a digital meter (ACCUCHECK™ glucometer and test strips). An individual was classified to have raised blood glucose/ diabetes mellitus based on random and fasting blood capillary glucose measurements cut-offs of 11.1 mmol/L and > 7.0 mmol/L respectively, or if they reported to have been previously diagnosed and informed to have diabetes by a health care worker and/or were taking medication for diabetes.

Total blood cholesterol levels were categorized either as ideal or high, with a cut off of point of 5.2 mmol/L or if an individual is currently on treatment for raised blood cholesterol. Low blood HDL cholesterol levels were categorized either as ideal or high, with a cut-off point of 1.17 mmol/L. A drop of blood from a finger prick was used to test for cholesterol levels using a digital meter (ACCUCHECK™ glucometer and test strips). A drop of blood from a finger prick was used to test for cholesterol levels using a digital meter (ACCUCHECK™ glucometer and test strips). Sugar intake was self-reported. High sugar intake was defined as regular consumption of excessive sugar in the form of sugary drinks and soda, processed foods high in sugar content on a regular, even daily, basis.

BMI was computed from height and weight measures, collected in centimeters and kilograms using SECA™ height boards and calibrated digital weigh scales respectively. Height was taken while the individual stood in an upright position on a flat surface. Obesity was diagnosed based on international guidelines recommended by the WHO expert consultation for 2008 [32]. General obesity was based on BMI cut-off of ≥30Kg/m^2^ and central obesity on waist circumference of > 94 cm for males and > 80 cm for females.

The other risk factors/variables were self-reported. Insufficient fruits and vegetables consumption is when an individual consumes less than the recommended five servings of fruits or vegetables daily. A serving of fruit/vegetables equates to one small fruit, ½ a cup of raw vegetables, one cup of leady greens or one banana. Insufficient physical activity is less than 150 h/week of moderate-intensive activity or less than 75 h/week of vigorous-intensive physical activities, which may include walking and cycling. An individual that spent 180 min/day sitting or reclining was also classified as physically inactive.

Unhealthy cooking fats referred to the use in cooking of saturated oils and solid fats e.g. lard, margarine, butter and vegetable fat for cooking, instead of unsaturated fats and oils. High salt intake was based on an individual reporting that they consumed salty and processed foods always or often, or if they added salt to cooked food. Excessive alcohol use was defined as the consumption of more than two standard drinks per day for females and more than three standard drinks for males. One standard drink contains about 14 g of pure alcohol, equivalent to 350 ml of beer with 5% alcohol by volume (alc/vol), or 1 glass table wine with 12% alc/vol. Current tobacco use was defined as the daily use of smoked or smokeless tobacco, in the form of cigarettes, cigars, pipes, snuff and other local tobacco products.

### Household wealth

A proxy index for wealth was created based on household-level variables including type of dwelling unit; ownership of the dwelling unit; construction materials of the dwelling unit specifically the roof and floor; source of cooking and lighting fuel; several household possessions/goods including a wall clock, electronics like radio and television, refrigerator, and furniture; source of water for drinking and cooking; and type of sanitation facility available for use by the household. A wealth index was then generated using principal component analysis (PCA), multivariate statistical technique. Principal components are weighted averages of the variables used to construct them. The generated index was then used to categorize the households into five categories (quintiles): poorest, second, middle, fourth and richest.

### Data analysis

The analysis was done using Stata 14.1 (Stata Corporation, College Station, TX). The outcome variable for this study is the number (count) of NCD risk factors in an individual in any combination out of the twelve described NCD risk factors. The number of NCD risk factors in an individual were categorised into three groups: 1–3, 4–6 and 7+ risk factors. Proportions and bar-graphs were used to summarize frequency/counts of NCD risk factors. Ordered logistic regression was used to assess the association between individual and household level sociodemographic factors with the presence of one or more NCD risk factors in an individual. Proportional odds assumption was tested using brant command method in Stata, with non-proportional odds model fitted wherever the assumption is violated.

We first fitted bivariate (unadjusted) ordered logistic regression models for each of the sociodemographic factors: age, sex, level of education, occupation, marital status, place of residence, ethnic group and household wealth-status with the outcome variable. We then fitted a multivariate model to assess the strength of association of each of the sociodemographic factors with the number of NCD risk factors in an individual. We used the likelihood ratio test to compare the goodness of fit, comparing a null model which is a special case of the alternative model with one additional variable being tested. Odds ratios are reported together with their 95% confidence intervals. Stratified analysis by sex was done to check for differences in the magnitude and pattern of the NCD risk factors between males and females, after confirming that there existed no statistical interactions between the sociodemographic variables in question with sex. Analysis was weighted at the individual level to account for study design, and to allow for generalizability of the findings.

### Ethical considerations

The study protocol was reviewed and approved by the Scientific and Ethics Review Unit at Kenya Medical Research Institute (Kemri) SSC No. 2607. Informed consent was sought from each and every participant prior to enrolement in the study. The study team was introduced to concepts of research ethics and encouraged to protect the rights of human research participants. Researchers upheld justice, regard for welfare and respect for study participants at all times during the study. During analysis and reporting, personal identifiers were delinked from the data.

## Results

### Characteristics of study participants

A total of 4066 individuals (51.4% females) were included in this analysis. This number was 90% of the 4500 individuals reached in the STEPs survey, from 4754 households that consented to participate in the study. Demographic characteristics of the study population are shown in Table 1. Forty-six percent of the study participants were under the age of 30 years. Overall, a small proportion of the study population (12.4%) did not possess any formal schooling, a proportion that comprised three times as many females as there were males, 18.0% vs 6.4%, respectively. Almost two in five individuals (38.2%) lived in urban areas. Individuals currently married or living together with a partner (i.e. in marital union) comprised 65.9% of the population, while 59.7% of study respondents were in some form of employment, the proportion of females being smaller (46.2%) compared to that of males (74.1%). There were more women compared to men in the lower wealth quintiles.

**Table 1:** Socio-demographic characteristics of study population

Indicator	Female	Male	Total	*P*-value
Wealth status				
Poorest	21.6	16.9	19.3	0.034
Second	22.2	20.6	21.5	
Middle	18.7	17.1	17.9	
Fourth	16.3	20.2	18.2	
Richest	21.2	25.2	23.1	
Age groups				
18–29	47.0	45.9	46.4	0.636
30–44	32.8	32.5	32.7	
45–59	15.1	16.4	15.7	
60–69	5.1	5.1	5.1	
Education level				
No schooling	18.0	6.4	12.4	< 0.001
Primary incomplete	23.9	23.3	23.6	
Primary complete	33.6	30.8	32.2	
Secondary school and higher	24.6	39.5	31.8	
Residence				
Rural	65.3	58.2	61.8	0.013
Urban	34.7	41.8	38.2	
Marital status				
Not in union	30.9	37.4	34.1	0.029
In union	69.1	62.6	65.9	
Ethnicity				
Kalenjin	13.2	16.4	14.8	0.001
Kamba	11.5	6.7	9.1	
Kikuyu	15.5	17.2	16.3	
Kisii	6.7	7.2	6.9	
Luhya	15.1	16.5	15.8	
Luo	11.2	10.2	10.8	
Meru	4.2	7.8	6.0	
Other~	15.8	14.4	15.1	
Somali	6.7	3.5	5.1	
Missing	0.0	0.1	0.1	
Occupation				
Employed	11.8	30.7	21.0	< 0.001
Self-employed	34.4	43.4	38.7	
Unemployed	53.8	25.9	40.3	
N	2467	1599	4066	

Data is weighted at individual participant level

~The ‘other’ethinc group comprises the Borana (22 (0.5%)), Embu (95 (2.1%)), Maasai (84 (1.9%)), Miji Kenda (182 (4.1%)), Turkana (106 (2.4%)) and unclassified category (404 (9.0%)) ethnic groups

**Fig. 1:**
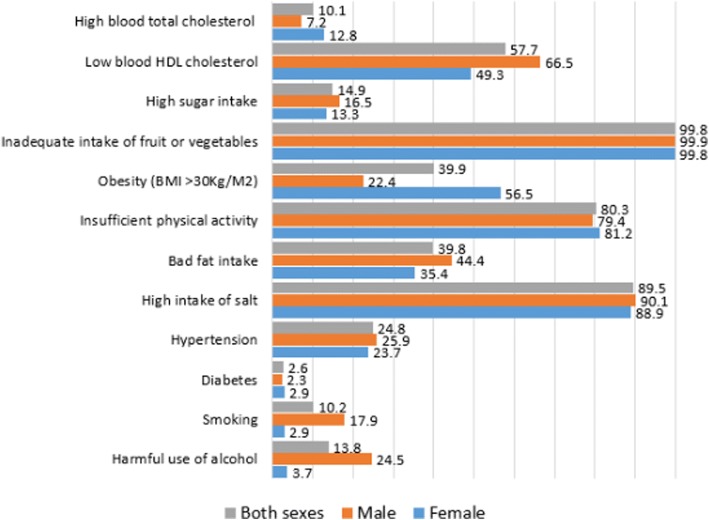
Prevalence of non-communicable disease risk factors in the study population

The weighted prevalence of the twelve NCD risk factors in this population is shown in Fig. 1. Overall (both sexes), the prevalence of insufficient consumption of fruits and vegetables, high salt consumption and insufficient physical activity were 99.8, 89.5, and 80.3% respectively. One in four individuals had high blood pressure while the overall prevalence of diabetes was 2.6%. Majority (80%) of individuals with raised blood pressure were new cases diagnosed in this survey, while about 50% of the individuals with raised blood glucose did not know their status before being diagnosed in this study.

Harmful alcohol use, daily tobacco use, hypertension, low high density lipoprotein (HDL) cholesterol, bad fat intake, and high sugar intake were more prevalent in males than females, 24.5% vs 3.7%, 17.9% vs 2.9%, 25.9% vs 23.7%, 66.5% vs 49.3%, 44.4% vs 35.4%, 16.5% vs 13.3%, while diabetes, obesity, and raised blood total cholesterol were more common among females, 2.9% vs 2.3%, 56.5% vs 22.4%, 12.8% vs 7.2%, respectively.

#### Occurence of non-communicable disease risk factors in the population

**Fig. 2:**
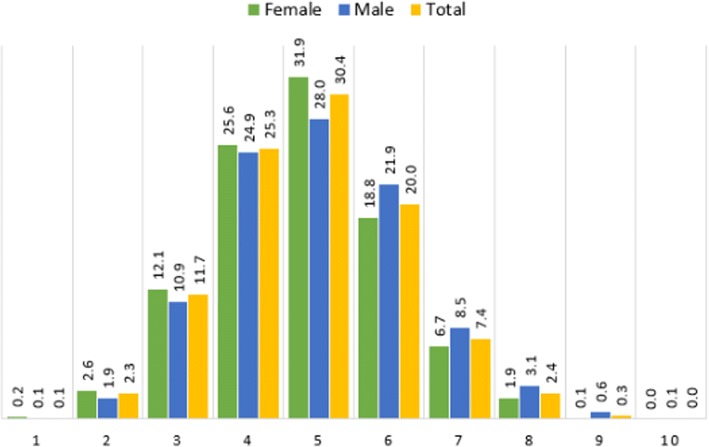
Distribution of non-communicable disease risk factors count in the study population

The highest number of risk factors recorded in an individual was ten out of the possible 12 NCD risks factors considered in this study. The bulk of the population (75.8%) possesed between four and six NCD risk factors. The biggest proportion, 30.4%, possessed five; 25.3% four; and 20.0% six NCD risk factors. A little less than 12% possesssed three, while only about 2 % possessed two or fewer NCD risk factors. About 10% possessed seven and more NCD risk factors. Figure 2 summarises the occurrence of the NCD risk factors, showing the differences between the sexes.

#### Prevalence of risk factor count by demographic characteristics

The number of NCD risk factors in individuals increased with their age, household wealth status, education, and employment but varied by ethnicity. Overall, men and women showed differences in the risk factor counts. Generally, men tended to have a higher number of NCD risk factors when compared to women. Whereas the NCD risk factor count increased among men by wealth status, education level, and occupation, these factors did not affect risk factor count among women. While women in union had a higher risk factor count compared to those not in union, marital status did not have any effect on risk factor count amont men. Participants in the younger age-groups, both male and female, were twice as likely to possess three or less NCD risk factors and thrice least likely to possess seven and more NCD risk factors compared the 45 years or older age-group. No differences were observed among urban and rural residents on the NCD risk factor counts. (Table 2).

**Table 2:** Distribution of NCD risk factor counts across population characteristics, STEPS survey (Both sexes, Male, Female)

Indicator	Both sexes				Male				Female			
	NCD risk factor count				NCD risk factor count				NCD risk factor count			
	1–3	4–6	7–10	p-value	1–3	4–6	7–10	p-value	1–3	4–6	7–10	*p*-value
Total	14.5	75.3	10.2		11.6	76.1	12.3		17.3	74.5	8.1	
Sex												
Female	17.3	74.5	8.1	0.002								
Male	11.6	76.1	12.3									
Wealth status												
Poorest	17.8	76.0	6.2	0.039	19.0	73.5	7.5	0.053	16.9	77.9	5.3	0.252
Second	13.9	77.8	8.3		11.9	79.0	9.2		15.7	76.8	7.5	
Middle	13.3	74.5	12.1		10.8	75.8	13.5		15.6	73.5	11.0	
Fourth	10.7	76.1	13.2		7.7	76.1	16.2		14.2	76.1	9.8	
Richest	16.4	72.4	11.3		10.1	75.7	14.2		23.5	68.6	7.9	
Age												
18–29	18.6	75.4	5.9	< 0.001	13.6	78.7	7.7	0.001	23.2	72.4	4.3	< 0.001
30–44	12.5	76.9	10.6		10.9	75.6	13.6		14.0	78.3	7.8	
45–59	8.6	71.3	20.1		7.1	70.9	22.1		10.3	71.7	18.0	
60–69	9.3	75.7	15.0		12.7	73.3	14.0		6.1	78.0	15.9	
Education												
No schooling	14.9	77.9	7.3	0.088	20.7	73.0	6.3	0.002	12.9	79.6	7.6	0.470
Primary school incomplete	14.4	74.5	11.1		13.1	72.7	14.2		15.6	76.1	8.3	
Primary school complete	13.6	78.5	7.8		8.9	83.4	7.7		17.8	74.3	8.0	
Secondary school and higher	15.4	71.6	13.0		11.3	72.9	15.8		21.7	69.7	8.6	
Residence												
Rural	14.1	76.5	9.4	0.368	12.1	77.4	10.5	0.245	15.8	75.8	8.4	0.388
Urban	15.3	73.3	11.5		10.8	74.3	14.8		20.3	72.1	7.6	
Marital status												
Not in union	17.7	73.2	9.1	0.061	13.1	77.7	9.3	0.163	22.9	68.1	9.0	0.013
In union	12.9	76.4	10.7		10.7	75.2	14.1		14.9	77.4	7.8	
Ethnic group												
Kalenjin	9.6	81.6	8.9	< 0.001	6.3	82.3	11.4	< 0.001	13.3	80.7	6.0	0.001
Kamba	20.2	73.8	6.0		7.9	84.2	7.9		27.0	68.0	5.0	
Kikuyu	7.9	75.4	16.7		5.6	76.2	18.3		10.3	74.7	15.0	
Kisii	20.6	72.9	6.6		16.6	74.5	9.0		24.7	71.2	4.1	
Luhya	12.4	78.6	9.0		8.5	79.2	12.3		16.4	78.0	5.6	
Luo	25.9	67.7	6.4		28.4	64.6	7.1		23.7	70.4	5.9	
Meru	6.6	71.1	22.3		6.3	74.2	19.5		7.0	65.7	27.3	
Other	15.4	75.5	9.1		13.8	74.6	11.6		16.8	76.3	7.0	
Somali	21.4	72.5	6.1		30.8	63.1	6.2		16.7	77.2	6.1	
Employment												
Employed	12.9	70.5	16.6	0.001	11.2	71.0	17.9	0.016	17.2	69.3	13.4	0.260
Self-employed	14.1	76.4	9.5		10.5	79.3	10.2		18.3	72.9	8.8	
Unemployed	15.8	76.8	7.4		13.9	76.8	9.3		16.7	76.7	6.5	

*NCD* Non-communicable diseases

### Factors associated with multiple non-communicable disease risk factors in Kenyan adult population

Table 3 shows individual and household level factors associated with an increased number of NCD risk factors in an individual, after adjusting for other factors. An increased number of NCD risk factors was associated with an individual’s age, sex, marital status and ethnic group. The odds of having a higher count of NCD risk factors increased from 1.62 to 2.64 from the age group of 30–44 years to age group of 60–69 years when compared to the younger people aged 18–29 years. This was common occurrence for both men and women.

**Table 3:** Factors associated with multiple NCD risk factors among adults in Kenya

Factors	Both sexes			Female			Male		
	1–3 vs. 4–6 & 7–10 factors			1–3 vs. 4–6 & 7–10 factors			1–3 vs. 4–6 & 7–10 factors		
	OR	LL 95%CI	UL 95%CI	OR	LL 95%CI	UL 95%CI	OR	LL 95%CI	UL 95%CI
Sex									
Female	1.00								
Male	1.45	1.13	1.87						
Age groups									
18–29	1.00			1.00			1.00		
30–44	1.62	1.25	2.11	1.67	1.16	2.40	1.62	1.10	2.39
45–59	3.20	2.10	4.86	3.57	2.35	5.43	2.98	1.58	5.62
60–69	2.64	1.83	3.82	4.45	2.98	6.67	1.59	0.87	2.91
Education level									
No schooling	1.00			1.00			1.00		
Primary school incomplete	1.09	0.74	1.60	0.99	0.63	1.57	1.35	0.65	2.82
Primary school complete	0.96	0.66	1.39	0.93	0.60	1.43	1.87	0.86	4.05
Secondary school and higher	0.79	0.48	1.29	0.87	0.43	1.77	1.26	0.57	2.77
Marital status									
Not in union	1.00			1.00			1.00		
In union	1.27	1.02	1.57	1.53	1.09	2.15	1.13	0.77	1.67
Ethnic group									
Kalenjin	1.00			1.00			1.00		
Kamba	0.45	0.29	0.72	0.43	0.21	0.86	0.62	0.35	1.10
Kikuyu	1.53	0.99	2.36	1.64	0.91	2.95	1.42	0.81	2.48
Kisii	0.51	0.30	0.88	0.53	0.26	1.10	0.48	0.24	0.96
Luhya	0.82	0.56	1.20	0.78	0.47	1.29	0.90	0.56	1.43
Luo	0.38	0.22	0.65	0.53	0.27	1.04	0.25	0.12	0.52
Meru	1.92	1.19	3.11	3.58	2.03	6.31	1.33	0.66	2.67
Other	0.78	0.49	1.23	0.87	0.44	1.69	0.70	0.40	1.22
Somali	0.59	0.24	1.46	0.87	0.35	2.15	0.38	0.14	1.01
Observations	4062			2464			1598		
1–3&4–6 vs. 7–10 Sec+									
Primary Incomplete	1.36	0.84	2.19				3.57	1.34	9.52
Secondary school and higher							3.40	1.31	8.85

Employment status, place of residence, and household wealth status were not significant in the model and were therefore not presented in this table

*LL* lower limit, *UL* upper limit, *CI* Confidence interval

The factors associated with an increase in NCD risk count in the population were mainly driven by the female sex, and thus there existed some differences in socio-demographic factors for a high count of NCD risk factors observed between men and women. Men were one and half times more likely to have higher counts for NCD risk factors compared to women. Being in a marital union was also associated with higher odds (1.27) of possessing higher counts of NCD risk factors. Women in a marital union were one and half times likely to have a higher count of NCD risk factors, but not so among men.

Overall (both sexes), compared to the Kalenjin ethnic group, the Kamba, Kisii, and the Luo had lower odds of having a higher counts of NCD risk factors (0.45, 0.51, 0.38 odds ratio respectively), while the Meru had almost twice as high odds (1.92) of having a higher count of NCD risk factors compared to the Kalenjin. Among men, being a Kisii or Luo was associated with lower odds (0.48 and 0.25 respectively) of having a higher counts of NCD risk factors. However, while a woman of the Akamba ethnic group had lower odds (0.43) of having a high NCD rik factors count, a women from the Meru ethnic group had 3.58 odds of possessing higher NCD risk factor counts when compared to the Kalenjin women.

## Discussion

Our findings reveal that up to 75% of the population in Kenya possessed between four and six NCD risk factors of any combination out of 12 assessed NCD risk factors: one in three of respondents possessed five NCD risk factors, a quarter possessed four risk factors, one in five respondents had six risk factors while about 12% possessed three NCD risk factors. The most prevalent NCD risk factors in the population, for both males and females, were insufficient consumption of fruits and vegetables, high salt consumption and insufficient physical activity, 99.8%, 89.5% and 80.3% respectively. Similar findings on low fruits and vegetable consumption were reported in a study conducted in a sub-sample of a Kenyan population [33], and in Bangladesh where more than 93% of the people consumed insufficient amounts of fruit and vegetables i.e. less than the recommended five servings/day [34]. A similar trend with a prevalence of over 70% for low fruit and vegetable consumption among men and women living in LMICs holds [35].

Some NCD risk factors were seemingly gendered. Harmful alcohol use, daily tobacco use, hypertension, low HDL cholesterol, high fat, and high sugar intake were more prevalent in males than females, while the opposite was true for diabetes, obesity, raised blood total cholesterol. These echo findings from other studies in Kenya [33], and in Malawi, which also showed tobacco smoking, alcohol drinking and raised blood pressure to be more prevalent in males compared to the females, with obesity and raised blood cholesterol was more common in females [36].

Although results from the World Health Survey on socioeconomic inequalities in risk factors for non-communicable diseases in LMICs showed that ‘physical inactivity was less prevalent in populations of low socioeconomic status, especially in low-income countries’ [37], and the global estimate for prevalence of physical inactivity among adults is 17% [6], we found very high levels (80.3%) of physical inactivity in the Kenyan population. Modern forms of transportation that include motorbikes both in urban and rural areas, as well as lack of spaces to exercise could contribute to inactivity in the Kenyan population.

NCD risk factors in individuals increased with age, marital status and ethnic group (more significantly among women) indicating that early screening would forestall the accumulation and effect on NCD risk factors in individuals. The association between NCD risk factor and ethnic grouping is not new, as a study conducted among minority groups in the USA reported a similar association [38]. We postulate that behavioural, cultural and societal factors that were not measured in this study, could account for these differences. Perhaps the socioeconomic opportunities accruing to married women or later in their reproductive life could explain the association between being in a marital union with an increased number of NCD risk factors in married women. It could also be that women in a marital union have other factors not measured in this study (such as the use of hormonal contraceptives or hormonal changes during their reproductive cycle) that may be associated with the accumulation of multiple NCD risk factors. Further research on how marital status, especially among women, is associated with multiple NCD risk factors is required to elucidate these findings.

The association between wealth and NCD risk factors has been reported in the World Health Survey [37]. Huge inequalities exist in the distribution and patterns of NCD risk factors across wealth quintiles, with current smoking and low fruit and vegetable consumption being more prevalent in the poorest wealth quintile than in the richest. Contrary to findings from Uganda, where rural dwellers were one and half times more likely to possess multiple NCD riks factors [39], whether one lived in urban or rural Kenya did not influence the number of NCD risk factors they possesssed. It seems therefore that no matter where one lives in Kenya, poverty and other social, physical and economic challenges provide little protection from NCDs risk factors. Poverty has been linked to the growth of the NCD burden: the poor, regardless of where they lived were disproportionately affected by the economic burden of the disease [40, 41] which also served to escalate poverty among them [42, 43].

### Strengths and limitations

Our findings suffer from one main limitation. Data on behavioural risk factors was based on self-reports and may be affected by potential under-reporting, especially on the socially discouraged practices like smoking/tobacco use and harmful alcohol consumption. That notwithstanding, our study applies a standard chronic disease risk surveillance approach (WHO STEPs) that confers comparability of our findings with those from other setttings and countries. Our findings provide new insights on the patterns and the distribution of multiple NCD risk factors together with their sociodemographic determinants nationally.

## Conclusions

The majority (75%) of the Kenyan adults possess between four and six NCD risk factors, while still a substantial proportion (10.1%) posses seven or more NCD risk factors. This is a clear indication of an impending NCD epidemic in this population that needs to be addressed. The epidemiological assessment of key NCD risk factors, their combination/clustering and distribution across differerent sociodemographic strata can inform the design of effective, targeted multisectoral interventions, especially those targeting behavioural and modifiable NCD risk factors, for the prevention of NCDs.

The WHO, in the *Global status report on non-communicable diseases 2010*, has ranked the monitoring and surveillance of risk factors a top priority to tackle growing NCD epidemics in low-resource settings like Kenya. More research is needed on perceptions of the Kenyan population regarding risk factors and the accompanying risk for developing NCDs, an aspect that influences individual level actions for the prevention and control of NCDs. Because the risk factors tend to cluster, and these risk factors act in an additive and a multiplicate way to cause NCDs, a ‘comprehensive approach’ rather than one based on a single factor is important to forestall cumulative effects of multiple NCD risk factors which occur over time [44]. Effective and multi-sectoral interventions are required to mitigate especially the behavioural and modifiable NCD risk factors in Kenya.

## Abbreviations

BMI: Body mass index
CVD: Cardiovascular diseases
HDL: High-density lipoproteins
LMICS: Low- and middle-income countries
NCD: Non-communicable diseases
PCA: principal component analysis
PDA: Personal digital assistant
SSA: sub-Sharan Africa
STEPs: WHO STEPwise approach to Surveillance (of NCD risk factors)
WHO: World Health Organisation
